# Nutritional Assessment of Children and Adolescents with Cancer in Various Resource Settings

**DOI:** 10.3390/cancers18050873

**Published:** 2026-03-08

**Authors:** Kunanya Suwannaying, Piya Rujkijyanont, Hiroto Inaba

**Affiliations:** 1 Leukemia/Lymphoma Division, Department of Oncology, St. Jude Children’s Research Hospital, 262 Danny Thomas Place Memphis, Memphis, TN 38105, USA; 2 Division of Hematology-Oncology, Department of Pediatrics, Faculty of Medicine, Khon Kaen University, Khon Kaen 40002, Thailand; 3 Division of Hematology-Oncology, Department of Pediatrics, Phramongkutklao Hospital and Phramongkutklao College of Medicine, Bangkok 10400, Thailand; 4 Department of Pediatrics, University of Tennessee Health Science Center, Memphis, TN 38163, USA

**Keywords:** nutrition, assessment, children, cancer, resource-setting

## Abstract

**Background:** Malnutrition has bidirectional effects in childhood cancer, as nutrition affects treatment-related adverse effects and outcomes. In turn, the cancer diagnosis and treatment, along with related psychosocial factors, can affect nutritional status. Nutritional evaluation is challenging because of the heterogeneous nutritional risks associated with a patient’s cancer diagnosis and socioeconomic status, as well as because of the variation in available resources and capacity in different global settings. **Methods:** This review summarizes methods for evaluating nutritional status and proposes a structured approach for use across different cancer types and resource settings. **Results:** Conventional anthropometric measures, including weight, height, and body mass index, along with longitudinal growth curve plotting using World Health Organization or Centers for Disease Control and Prevention growth charts, are widely used but may not adequately detect changes in body composition. In resource-limited (limited-access) countries, where equipment and trained personnel are lacking, history taking, physical examination, and anthropometric measurements should be prioritized, along with basic body composition measures such as mid-upper arm circumference. In partial-access settings, biochemical assessments and bioelectrical impedance analysis may be added to identify micronutrient deficiencies and changes in lean and fat mass, respectively. In full-access settings, advanced body composition imaging techniques (e.g., dual-energy x-ray absorptiometry, computed tomography, and magnetic resonance imaging) can be incorporated. The approaches should also be adjusted based on the cancer diagnosis and treatment. **Conclusions:** Tailoring nutritional assessment strategies across diverse resource settings and diagnoses would be beneficial for targeted interventions that may improve clinical outcomes. Further research, quality improvement studies, and policy-level initiatives are necessary to develop effective assessments.

## Simple Summary

Nutrition has bidirectional effects in children with cancer. It influences, and is influenced by, the disease and treatment, starting from the diagnosis and continuing through therapy into survivorship. Therefore, a longitudinal comprehensive nutritional assessment is essential to define the patient’s nutritional status and to guide management. Assessment strategies should be tailored not only to specific cancer types but also to the available health care resources. In this review, we discuss practical methods for evaluating malnutrition, including their advantages and limitations. We also provide a structured approach for use in various resource settings. This approach will guide nutritional management that can enhance treatment outcomes for children with cancer.

## 1. Introduction

Malnutrition, defined as “deficiencies, excesses, or imbalances in a person’s intake of energy and/or nutrients” and referring to both undernutrition and overnutrition, is a major global health issue for children [1,2]. Undernutrition is more common in low- and middle-income countries (LMICs) than in high-income countries (HICs), with prevalences of 35.8%, 22.8%, and 4.0% in low-income countries (LICs), middle-income countries (MICs), and HICs, respectively, in 2024 [2]. Conversely, the main issue in HICs is overnutrition, with the recent increases in obesity prevalence among US children rising from 5.2% in 1971–1974 to 19.3% in 2017–2018 [3].

Both under- and overnutrition occur in children with cancer (Supplementary Table S1) [4,5,6,7,8,9,10,11,12,13,14,15]. Undernutrition is common at diagnosis, particularly in LICs (29.3–30.5%) and MICs (8.1–45.6%). In HICs, the reported prevalences of undernutrition and overnutrition at diagnosis were 5.8–17.4% and 7.0–20.7%, respectively. As more than 80% of new childhood cancer cases are diagnosed in LMICs, this underscores the importance of resource-appropriate strategies to identify malnutrition [16].

Traditional anthropometric measurements such as weight, height, and body mass index (BMI) (weight [kg]/height [m]^2^) have been used to identify nutritional issues (Table 1). Body composition analysis and biochemical analysis can enhance the evaluation. This article reviews the unique nutritional status of pediatric patients with cancer and various assessment methods ranging from basic to advanced techniques, and it provides recommendations for detecting malnutrition across resource settings.

## 2. Undernutrition in Children with Cancer

**Figure 1:**
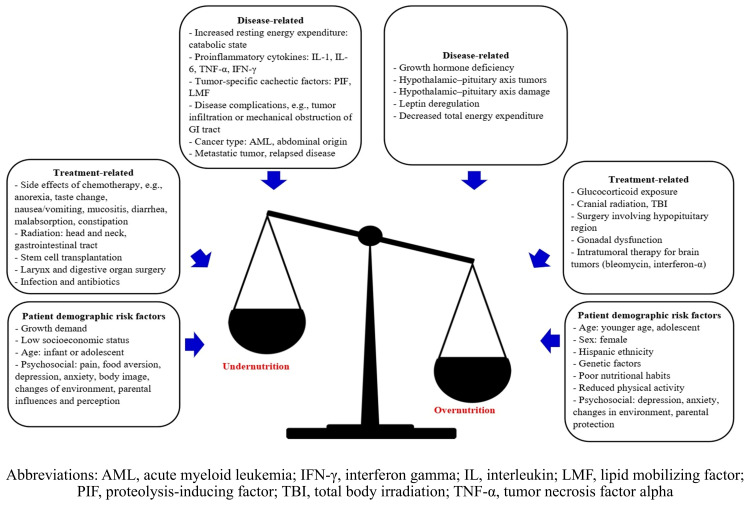
Factors related to undernutrition and overnutrition in children with cancer.

Multiple factors contribute to undernutrition, including cancer diagnosis, staging, treatment regimens, alterations in metabolism, and socioeconomic and psychological status (Figure 1). Acute myeloid leukemia, tumors involving the gastrointestinal tract, advanced-stage solid tumors, hematopoietic stem cell transplantation, and relapsed disease are associated with undernutrition [20]. Intensive chemotherapy contributes to anorexia, nausea, vomiting, mucositis, and malabsorption, and some agents have distinctive side-effects, such as the associations of asparaginase-induced pancreatitis and vincristine-related neuropathy with sarcopenia [20]. Radiation therapy (RT), especially when combined with surgery of the head, neck, and gastrointestinal tract, can affect eating and swallowing. Pro-inflammatory cytokines, such as interleukins 1 and 6 and interferon gamma, contribute to anorexia and a catabolic state, produce acute-phase reactant proteins (e.g., C-reactive protein, fibrinogen), and enhance protein turnover [20]. Low socioeconomic status increases the probability of malnutrition, potentially due to limited access to food and education [21]. Weight loss, reduced activity, and loss of independence can cause emotional distress in patients, whereas caregivers often feel helpless when expectations about increased intake are unmet. Differences in perspectives between patients and caregivers may negatively affect their relationships and coping behaviors [22]. Cultural stigma and misconceptions about cancer and food may further contribute to negatively affecting eating behavior through unnecessary dietary restrictions and inadequate protein/energy intake [23,24].

Undernutrition alters the pharmacokinetics of chemotherapy by decreasing liver oxidative functions (e.g., cytochrome P450 activity) and reducing renal blood flow, glomerular infiltration, and tubular secretion as a result of protein deficiency, leading to reduced clearance of drugs such as methotrexate and vincristine and increased adverse effects [25]. Undernutrition impairs both innate and adaptive immune functions, cytokine production, antibody response, and mucosal integrity as a result of deficiencies in macronutrients (e.g., protein and fatty acids) and micronutrients (e.g., vitamin A and zinc) [26].

## 3. Overnutrition in Children with Cancer

Overnutrition in children with cancer is influenced by patient demographics, diagnosis, treatment, diet, and activity levels. It often occurs during therapy and continues into survivorship [9,15]. For childhood cancer survivors, incorporating specific polygenic risk scores with treatment and lifestyle factors improves prediction models for severe obesity [27].

Glucocorticoids increase appetite, energy intake, cellular lipid accumulation, sarcopenia, and insulin resistance [15]. Chemotherapy-related fatigue and protective parental permission often lead to a sedentary lifestyle, contributing to increased fat tissue and decreased muscle mass [28].

Chemotherapy pharmacokinetics are altered by extending the half-life (e.g., of cyclophosphamide, doxorubicin, or methylprednisolone) or by enhancing clearance (e.g., of methotrexate and 6-mercaptopurine), which lead to over-dosing and under-dosing, respectively [29]. Therefore, pharmacokinetic-based dosing may offer optimal treatment [30,31]. Overnutrition also impairs health-related quality of life, particularly in the emotional and social domains, as affected children tend to experience increased fear, sadness, anger, social difficulties, and cognitive challenges [32]. After therapy, the incidence of obesity in survivors remains high, with potential for premature adiposity rebound, especially in patients with acute lymphoblastic leukemia (ALL) who have received steroids or cranial RT (CRT) [15,33]. Lower energy expenditure, physical inactivity, and unhealthy dietary habits during treatment tend to persist post therapy [11,15] and are associated with increased risks of metabolic and cardiovascular diseases [34].

## 4. Evaluation of Nutritional Status

Comprehensive assessment includes history assessment, dietary evaluation, biochemical evaluation, and anthropometric and body composition measurements. The optimal nutritional evaluation may vary based on the resources available.

## 5. History Assessment and Dietary and Nutritional Evaluation

History assessment and dietary evaluation are simple and effective methods across all resource settings. The assessment should include a medical and nutritional history, prior growth status, cancer type and treatment, surgical history, treatment side-effects, supportive/complementary therapy, dietary intake, feeding environment (e.g., food choices, accessibility, policy, promotion, and habits), socioeconomic status, and physical activities.

In resource-limited settings where few dietitians or physicians are available, the Screening Tool for the Assessment of Malnutrition in Paediatrics (STAMP), the Screening Tool for Risk on Nutritional Status and Growth (STRONGkids), and the nutrition screening tool for childhood cancer (SCAN) can help identify patients at risk for malnutrition (Table 1) [17,18,19]. SCAN was specifically developed for use with children with cancer. These methods primarily screen for undernutrition rather than overnutrition and are more applicable at diagnosis and during therapy than for survivors, because some questions concern active disease and treatment.

Assessing dietary intake is necessary to evaluate the adequacy of calories, macronutrients (carbohydrates, fat, and proteins), and micronutrients (minerals and vitamins). The assessment employs 24 h recall, food frequency questionnaires, a food diary, and a remote food photography method [20]. Nutritional information should be collected systematically to ensure patients meet the Recommended Dietary Allowances for optimal growth [20]. Because of the increased catabolic state and potential gastrointestinal or renal losses in patients with cancer, protein and energy requirements should be adjusted individually. Signs of malnutrition and vitamin or mineral deficiencies, such as subcutaneous fat loss, muscle wasting, changes in skin and hair, fluctuations in weight, edema, and evidence of nutrient deficiencies, should be assessed (Supplementary Table S2) [35]. Conditions that affect nutritional status, including difficulty chewing and swallowing, mucositis, vomiting, diarrhea, constipation, flatulence, belching, and indigestion, are also important [36].

## 6. Biochemical Evaluation

Biochemical measurements provide additional nutritional data on protein status (via serum albumin, pre-albumin, and transferrin levels), organ function (via blood urea nitrogen, creatinine, liver enzyme, and bilirubin levels), metabolic status (via electrolyte and blood sugar levels and a lipid profile), and micronutrient deficiencies [36]. Serum albumin and pre-albumin measurements are commonly used but can be affected by systemic inflammation, liver function, fluid balance, and medications such as asparaginase and steroids [14]. Patients with rapid weight loss or severe undernutrition may develop refeeding syndrome, requiring frequent monitoring of basic biochemistry, including serum glucose, urea, creatinine, potassium, phosphate, calcium, albumin, and magnesium levels [20]. Combining clinical examinations with laboratory tests can enhance the detection of malnutrition and of certain micronutrient deficiencies [35], although some tests are not available in resource-limited settings.

## 7. Anthropometric Measurements: Measurements of Weight, Height, and Body Mass Index

Weight, height, and BMI for age and sex are conventionally used to classify the nutritional status of children in both LMICs and HICs. Longitudinal growth curves based on World Health Organization (WHO) or Centers for Disease Control and Prevention (CDC) growth charts have been widely used (Table 2) [37]. WHO growth charts categorize undernutrition as wasting (thinning), stunting, and underweight, whereas overnutrition is classified as overweight and obesity, using the parameters shown in Table 2 [38]. CDC charts use weight for a given length to define underweight and overweight for children aged 0–3 years, and they use BMI to define underweight, overweight, and obesity for children aged 2–20 years [39].

WHO growth standards for children under 5 years were developed from six diverse countries/regions (Brazil, Ghana, India, Norway, Oman, and California) [38]. The charts for children aged 5–19 years were generated from the 1977 National Center for Health Statistics curve in 22 countries and merged with those for children younger than 5 years [38]. The values of +1 standard deviation (SD) and +2 SD of the WHO BMI for age Z-score at 19 years of age correspond to the adult overweight cut-off (≥25 kg/m^2^ to <30 kg/m^2^) and obesity cut-off (≥30 kg/m^2^), respectively [40]. However, the cut-offs for overweight and obesity differ for children younger than 5 years (being >2 SD to ≤3 SD and >3SD, respectively) and those aged 5–19 years (being 1 SD to ≤2SD and >2SD, respectively), although some studies apply the same criteria for overweight and obesity (>2 SD to ≤3 SD and >3 SD, respectively) across all ages [7,14]. The WHO adopted a cautious approach to avoid restrictive diets in young children, who are still growing, and because of limited data on the functional significance of the cut-offs for the upper end of the BMI-for-age distribution [42].

The CDC charts reflect the US growth pattern during 1963–1994 and may not represent the current population, which has an increased prevalence of obesity [3]. They are widely used for US children aged 2–19 years, with weight, height, and BMI parameters being presented as percentiles [43]. Extreme values (e.g., the ≤3rd and ≥97th percentiles) are difficult to interpret and can be converted to Z-scores, [44]. Lower or higher percentile values indicate malnutrition, with underweight (<5th percentile), overweight (≥85th to <95th percentile), and obesity (≥95th percentile) corresponding to Z-scores of <−1.645, ≥1.036 to <1.645, and ≥1.645, respectively.

WHO charts are recommended over CDC charts for use in resource-limited countries as they use leaner control samples, thereby minimizing the underestimation of obesity. For overnutrition, as the cut-offs for overweight and obesity differ for children younger than 5 years and those aged 5 years or older, Z-scores are beneficial for longitudinal assessments across age and sex [44]. Alternatively, clinicians should evaluate the two age groups (<5 years vs. ≥5 years) separately [42]. The CDC charts are more appropriate for US children, as the WHO criteria can underestimate undernutrition. The WHO growth chart standards are recommended for children younger than 2 years because they reflect optimal physiological growth, especially in breast-fed infants [41].

When patients are evaluated, their socioeconomic and cultural background can differ markedly from that of the populations evaluated in WHO and CDC growth curves, for which data were collected more than three decades ago [45]. If local normative values are unavailable, we can still use the WHO and/or CDC growth curves, but it is better to have growth curves for local healthy control populations with similar socioeconomic and cultural backgrounds that can be used to compare the status of pediatric oncology patients. Such comparisons may be used to evaluate patients across diverse global populations.

## 8. Body Composition Analysis

Body composition analysis estimates the quantity and differentiation of tissue compartments, i.e., the fat mass (FM) and fat-free mass (FFM) (or lean mass [LM]), which BMI cannot assess. The FM is further classified as visceral adipose tissue (VAT) or subcutaneous adipose tissue (SAT) (Table 3) [20].

## 9. Anthropometric Body Composition Evaluation

### 9.1. Waist Circumference, Waist-to-Hip Ratio, and Waist-to-Height Ratio

VAT is strongly associated with metabolic syndrome and cardiovascular risk [48]. Waist circumference (WC) is used to estimate VAT because of its strong association with abdominal girth [48]. WC is measured, to the nearest 0.1 cm, midway between the lowest rib and the superior border of the iliac crest at the end of an expiratory breath. International age- and sex-specific WC percentiles are available for healthy children and adolescents (aged 6–18 years), with measurements above the 90th percentile being linked to higher cardiovascular risk [49]. The waist-to-hip ratio (WHR) and waist-to-height ratio (WHtR) are alternative parameters. The WHR, with a ratio above 0.90 in adult males and 0.85 in females, respectively, increases cardiometabolic risks [50]. The WHtR with cut-offs at or above 0.5 predict cardiometabolic risk regardless of age, sex, or ethnicity [51].

Waist-related measures should be used with caution in patients with an abdominal mass, organomegaly, or ascites and in those undergoing abdominal RT.

### 9.2. Mid-Upper Arm Circumference

Mid-upper arm circumference (MUAC) is measured at the mid-point between the acromion and the olecranon process of the non-dominant upper arm (typically the left one). It represents the sum of upper-arm muscle and subcutaneous fat. MUAC is recommended for undernutrition screening because the upper arm is typically unaffected by abdominal mass, amputation, or edema [36]. In childhood cancer studies, MUAC correlated positively with BMI and could predict FFM, as validated by bioelectrical impedance analysis (BIA) and dual-energy X-ray absorptiometry (DXA) [52,53,54]. The WHO developed age- and sex-based MUAC references for children aged 3–60 months, as well as cut-offs for those aged 5–14 years [55,56]. The cut-offs for undernutrition are <12.5 cm for <5 years, <15.5 cm for 5–9 years, and <18.5 cm for 10–14 years. For severe undernutrition, the cut-offs are <11.5 cm for <5 years, <13.0 cm for 5–9 years, and <16.0 cm for 10–14 years [56]. The color-coded children’s MUAC measuring tape, created by the United Nations Children’s Fund (UNICEF), with a cut-off point of 11.5 cm for severe malnutrition, enables rapid assessment of nutritional status in children aged 6–59 months [35]. The WHO also constructed MUAC-for-age Z-score references for ages 5–19 years [57]. MUAC and skinfold percentiles were derived from the United States National Health and Nutritional Examination Survey (NHANES) data (representing 19,097 individuals aged 1–74 years) [58]. The Asociación de Hemato-Oncología Pediátrica de Centro America (AHOPCA) recommends age- and sex-specific MUAC cut-offs of less than the 10th and 5th percentiles for undernutrition and severe undernutrition, respectively [59]. In hospitalized children, MUAC Z-scores correlate well with the MUAC cut-offs and are more sensitive for detecting severe malnutrition [60].

### 9.3. Skinfold Measurements

Skinfold thickness serves to estimate FM because most body fat is stored subcutaneously [33]. The common measurement sites are the triceps and subscapular areas. Triceps skinfold thickness (TSFT) is measured at the same location as for MUAC. Subscapular skinfold thickness (SSFT) is measured 2.0 cm medial to and above the inferior angle of the scapula. The WHO provides TSFT references for children aged 3–60 months but lacks specific cut-offs [55]. Body fat in overweight and obese patients can be calculated using TSFT with the NHANES equations and age- and sex-specific cut-offs: values below the 10th percentile correspond to low body fat, the 10th–90th percentile corresponds to normal body fat, and the 90th percentile or higher corresponds to high body fat [58].

### 9.4. Use of Anthropometric Body Composition Evaluation for Pediatric Patients with Cancer

Arm anthropometry is more sensitive than BMI for detecting undernutrition, especially in patients with an abdominal mass or altered hydration status (e.g., edema, effusion, or ascites) [5,13]. Positive correlations between MUAC and FFM and between TSFT and FM were confirmed by DXA in children with cancer and in survivors [33,52]. Studies in patients with various childhood cancers have shown that FFM by MUAC decreases and FM by TSFT increases during therapy, consistent with the BIA findings [53]. Experiences from LMICs and HICs have revealed that anthropometric body composition evaluation is feasible and effective (Supplementary Tables S3 and S4). For MUAC and TSFT, WHO Z-scores or NHANES percentiles/Z-scores in children younger than 5 years and NHANES percentiles/Z-scores for those older than 5 years are recommended for accurate nutritional assessment and for longitudinal monitoring. Although the WHO reference for children older than 5 years still needs validation [4,21], the WHO cut-offs are beneficial for screening and are more effective than BMI alone for children younger than 5 years in resource-limited countries.

Waist measurements are studied primarily in childhood cancer survivors to identify those with obesity and/or cardiometabolic risks [18,33]. WHtR is more sensitive than BMI for detecting a high total fat percentage, as has been validated by DXA [33]. Both skinfold and waist measurements can help predict future cardiovascular and metabolic outcomes in obese survivors [33,34].

## 10. Advanced Body Composition Evaluation

### 10.1. Bioelectrical Impedance Analysis

BIA is a simple, non-invasive, and inexpensive technique that uses electrical current conductor activity to differentiate FM from FFM; FFM and body water have higher conduction than FM [20,61]. A systematic review found that BIA has excellent reproducibility in estimating FM and FFM, with strong correlation to reference methods, although BIA may underestimate FM [61].

### 10.2. Dual-Energy X-Ray Absorptiometry

DXA uses the differential low-dose x-ray absorption properties of bone mineral, FM, and FFM (LM) [20]. The NHANES published an age- and sex-specific DXA dataset for the healthy US population (aged 8–85 years), with regional reference values for the arm, leg, and trunk [62,63]. Parameters for children include the total body fat percentage, total LM, height-adjusted LM, bone mineral density (BMD), and bone mineral content [62]. In pediatric studies, FM and FFM are often corrected for height [m]^2^ to derive the FM and FFM indices, enabling Z-scores to be computed based on age- and sex-specific reference data [62,63,64]. DXA provides accurate and reproducible data with low cost, short test duration, and minimal radiation exposure [20,46]. DXA can indirectly estimate VAT and SAT from total body and trunk fat measurements, but discrepancies among instruments and software have been reported [65].

### 10.3. Computed Tomography and Magnetic Resonance Imaging

Computed tomography (CT) and magnetic resonance imaging (MRI) are used to diagnose and monitor malignant disease, especially in the chest and abdominal area. They are the gold standards for body composition analysis, providing 3-dimensional images to differentiate LM, SAT, and VAT and to quantify intramuscular adipose tissue, which cannot be effectively evaluated by other methods [46,47,66]. VAT and intramuscular adipose tissue are associated with insulin resistance and metabolic syndrome [67]. An intermodal study comparing single-slice CT and MRI showed high correlations for both skeletal muscle area and muscle fat infiltration [68]. Apart from BMD measurement, single-slice quantitative CT can measure body composition with high accuracy and low radiation exposure [28]. CT from T10 to S1 to measure intrabdominal adipose tissue in adults showed that single images correlated strongly with total volumes for VAT (R^2^ = 0.65 − 0.96) and SAT (R^2^ = 0.80 − 0.96) [69]. VAT at L1/L2 or T12/L1 is associated with a greater risk of metabolic syndrome than VAT at other levels. To define sarcopenia in children, the abdominal skeletal muscle area (SMA) (at the intervertebral space between T12 and L1 and at the L3 level) and the total psoas muscle area (tPMA) (at the intervertebral space between L3 and L4 and at the L4 and L5 levels) can be measured [70,71,72]. Age- and sex-specific reference data are available for SMA in the US population aged 2.0–18.9 years and for tPMA in Canadian children aged 1–16 years [73,74]. There are no standardized criteria for diagnosing sarcopenia in children, but some reports have used tPMA Z-score cut-offs below −1 or −2 [70,71].

MRI can measure LM, SAT, and VAT, and it provides more soft tissue information than CT, including data on muscle edema, inflammation, atrophy, and fatty infiltration [66]. However, MRI is expensive, less accessible, and time-consuming, and may require sedation of young children. Age- and sex-specific SAT and VAT reference charts for children aged 6–18 years are available for Caucasian populations [75].

### 10.4. Use of Advanced Body Composition Evaluation for Pediatric Patients with Cancer

Nutritional studies in HICs have focused on body composition, using advanced technologies (Supplementary Tables S3 and S4). Studies using BIA in children with various cancers have shown that BMI and FM increase during therapy, whereas FFM declines [53,76]. DXA has demonstrated greater efficacy than BMI in detecting obesity and sarcopenia in childhood cancer survivors [33,77,78]. CT for diagnosis and disease monitoring in children with cancer, such as those with lymphoma or solid tumors, has proved beneficial for detecting increased FM and decreased LM during and after treatment [28,70,71,72,79]. In children with neuroblastoma, pre-operative sarcopenia has been associated with worse 5-year survival after surgery [70].

## 11. Cancer Diagnosis-Specific Considerations in Nutritional Assessment

Nutritional impairment in childhood cancer varies according to the disease biology and treatment exposure. Therefore, assessment should be tailored individually, based not only on the patient characteristics but on the specific cancer diagnosis and treatment strategy. In ALL, patients often shift from undernutrition at diagnosis to overnutrition during and after therapy, largely driven by glucocorticoid effects and reduced physical activity, which starts as early as during induction therapy [15,79]. Body composition studies show that BMI changes correlate with increased SAT and VAT and with reduced LM, consistent with sarcopenic obesity [28]. As weight extremes (both underweight and overweight/obesity) during therapy are associated with higher incidences of adverse effects (infection, requirement for hematological and nutritional support, prolonged treatment courses, and poorer performance status) and inferior survival outcomes [9,80], nutritional assessment should start during the early phase of therapy (e.g., at diagnosis and/or during induction therapy) to guide prompt intervention.

Children with AML commonly experience weight loss and a decline in BMI Z-score during intensive induction therapy, whereas obesity may emerge during survivorship [11]. Similar to patients with ALL, overweight/obesity and underweight in patients with AML have been associated with worse survival outcomes and increased treatment-related mortality [81,82]. Therefore, nutritional assessment in AML should prioritize the prevention and early detection of, and intervention for, treatment-related weight loss, as well as enhanced monitoring to guide supportive care for both overweight/obese and underweight patients. In survivors, monitoring for weight gain and adverse body composition changes is important.

Children with solid tumors often experience systemic inflammation, oxidative stress, inadequate calorie intake, physical inactivity, and, in some cases, sarcopenia due to delayed diagnosis [71]. Weight and BMI often decline during the first few months after diagnosis, and greater declines in LM during therapy have been associated with higher relapse rates and poorer overall survival [71,83]. BMI and weight status may improve during less intensive treatment phases; however, these measures may not fully capture changes in LM and VAT status [72,76,84]. Therefore, when feasible, arm anthropometry or imaging-derived body composition measures should be incorporated to better identify nutritional risk and guide early intervention.

For children with brain tumors, weight and BMI often increase rapidly as height growth declines. These changes are accompanied by higher FM and lower FFM [76]. FFM is frequently reduced at diagnosis and remains low throughout treatment [76]. These patterns can be attributed to endocrinopathy, such as growth hormone (GH) deficiency due to the tumor location, CRT, or surgery, as well as reduced physical activity as a consequence of neurological impairment. Untreated GH deficiency results in decreased FFM, muscle weakness, lower energy expenditure, and poor exercise tolerance, leading to long-term obesity and cardiometabolic disease [85]. Therefore, nutritional assessment in this population should focus on longitudinal monitoring of weight, height, and BMI, along with assessments of food intake and physical activity, body composition analysis, neurofunctional evaluation, and hormonal measurement.

## 12. Practical Recommendations for Various Resource Settings

**Figure 2:**
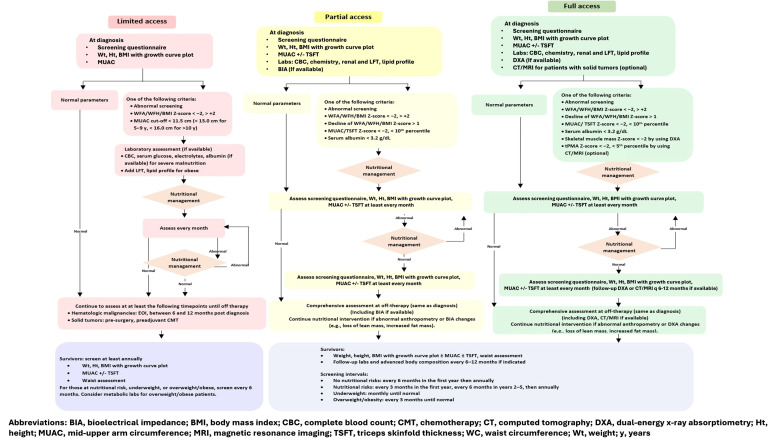
Proposed nutritional assessment algorithm for children with cancer (based on available resources).

We recommend tailored nutritional assessment across different resource settings, categorized as limited-access, partial-access, or full-access based on the availability of biochemistry evaluation, anthropometric and advanced body composition evaluation, and nutritionist support (Table 4 and Figure 2). In limited-access settings, where weight, height, and BMI with growth curve plots are the primary assessment tools, incorporating screening tools such as SCAN with MUAC can improve sensitivity in detecting undernutrition, especially in patients with conditions that might make BMI misleading, such as amputations or large tumor masses. In cases of severe malnutrition, biochemical evaluation before and during nutritional management is recommended to prevent refeeding syndrome.

For partial-access settings, we recommend using assessments similar to those used in limited-access settings but adding biochemical evaluations and body composition assessments such as BIA and evaluating longitudinal changes. This enables the early detection of adiposity and sarcopenia resulting from treatment, thereby helping to identify patients who may benefit from additional interventions. If assessing every patient is not feasible, priority should be given to those with abnormal BMI (whether due to undernutrition or overnutrition), sarcopenia, osteopenia, abnormal cardiometabolic profiles, or exposure to treatments that increase metabolic risk (e.g., corticosteroids and CRT).

In full-access settings, DXA is preferred for estimating body composition and BMD. CT and MRI are also preferred for patients with solid tumors; they provide accurate measurements of body composition alongside disease staging and treatment response evaluation, although standard references for these measurements require further study.

Nutritional assessment should continue into survivorship, when nutritional problems can persist and increase cardiometabolic risk. For childhood cancer survivors in limited-access settings, annual anthropometric measurements are recommended, with more frequent follow-ups and laboratory tests for those with nutritional issues. In partial-access and full-access settings, we recommend combining risk-based anthropometric assessments with occasional laboratory tests (every 3–6 months) and body composition monitoring (annually). The follow-up frequency in the first year should depend on the risk and can be reduced if the patient is clinically stable. If no risk is present, follow-up should be conducted every 6 months during the first year then annually thereafter. Patients with risk factors such as poor eating habits, inactivity, hyperlipidemia, decreased LM, increased FM, or underweight or overweight/obesity should undergo more frequent monitoring until the condition resolves.

## 13. Limitations and Future Directions

The lack of validation studies in LMICs may limit the applicability of our recommendations, especially because of the variability in anthropometric profiles based on ethnicity and race, the diversity of socioeconomic status and its influence on nutritional status, and the lack of economic feasibility analyses for cost-effectiveness. Economic feasibility analyses have focused on nutritional intervention rather than on assessment tools and strategies. However, nutritional assessment has been shown to be feasible in LMICs by combining the interpretation of anthropometric (e.g., height, weight, MUAC, and TSFT), biochemical, clinical, dietary, and economic data [86]. Furthermore, although advanced imaging techniques are resource-intensive, opportunistic analysis of CT or MRI scans obtained for routine disease monitoring may provide a feasible approach without incurring substantial additional cost or radiation exposure [70,71,72,79].

Implementing nutritional assessment programs for children with cancer, particularly in LMICs, also faces several challenges, including limited training of healthcare professionals, low awareness of the importance of nutrition in cancer care, a shortage of dietitians, and insufficient resources such as validated screening tools and standardized data collection systems [87,88,89]. Providing training and education for multidisciplinary health care professionals is essential to improve the early identification of malnutrition. In addition, research and quality improvement initiatives can support the implementation of standardized nutrition screening and referral pathways [89]. Policy-level engagement from stakeholders is also important to increase resource allocation.

Although this article does not address the management of nutritional status, the committee on Pediatric Oncology in Developing Countries (PODC) of the International Society of Pediatric Oncology (SIOP) has published a framework that includes nutritional resources and services that are feasible and safe for each level of infrastructure, thereby providing guidance for optimal nutritional care [35].

## 14. Conclusions

Nutrition is a crucial component of pediatric oncology care, affecting both short-term and long-term outcomes. Nutritional assessment must be tailored to available resources. Our recommendations aim to provide a structured approach to evaluating nutritional status, ranging from basic anthropometry to advanced body composition techniques. Further research is essential to develop nutritional care guidelines across diverse settings.

## Figures and Tables

**Table 1:** Common nutritional screening tools used with pediatric populations [17,18,19].

Screening Tools	SCAN	STAMP	STRONG_kids_
Target population	Children with cancer	General hospitalized children	General hospitalized children
Validation studies available for specific pediatric cancers and timepoints?	Yes, cross-sectionally evaluated at diagnosis, active treatment, maintenance, post therapy	No	Yes, no specific time point
Components	6 questions	2 questions and anthropometric data	4 questions
Criteria used	Risk of cancer Treatment intensity GI tract symptoms History of poor intake History of weight loss Signs of undernutrition	Diagnosis Dietary intakes Weight and height	Underlying disease with risk of malnutrition Signs of poor nutritional status Diarrhea, poor intake or history of nutritional supplement History of weight loss or stunted growth
Score indications	≥3 points: at risk of malnutrition	4–5 points: high risk 2–3 points: medium risk 0–1 point: low risk	4–5 points: high risk 1–3 points: medium risk 0 point: low risk
Recommendations	≥3 points Refer to dietician for further assessment	High risk Refer to nutritional supportteam Medium risk Monitor nutritional intake for 3 days and repeat exam after 3 days Low risk Routine clinical care	High risk Consult clinician and dietician for full diagnosis and individual nutritional advice Medium risk Consider nutritional intervention Low risk No nutritional intervention necessary
Pros and cons	Pros Designed specifically for childhood cancers Enables easy screening without anthropometric measurement Cons Need data on cancer risk and treatment intensity	Pros Usable by any health care provider without specific oncology knowledge Cons Screens all cancers as at least medium risk; cannot distinguish malnutrition patients	Pros Usable by any health care provider without specific oncology knowledge Enables easy screening without anthropometric measurement Cons Screens all cancers as at least medium risk; cannot distinguish malnutrition patients

Abbreviations: GI, gastrointestinal; SCAN, the nutrition screening tool for childhood cancer; STAMP, Screening Tool for the Assessment of Malnutrition in Paediatrics; STRONG_kids_, Screening Tool for Risk on Nutritional Status and Growth.

**Table 2:** Anthropometric classifications of malnutrition [38,39,40,41].

WHO Criteria				CDC Criteria			
Age	Nutritional Status	Indicator	Cut-Off Points	Age	Nutritional Status	Indicator	Cut-Off Points
Undernutrition							
0–5 y	Wasting (thinness)	W/L or W/H or BMI	≤−2 SD	0–2 y	Undernutrition	Recommend using WHO growth standards	
	Stunting	L/A or H/A	<−2 SD				
	Underweight	W/A	<−2 SD				
5–19 y	Thinness	BMI	<−2 SD	2–19 y	Underweight	BMI	<5th percentile
	Stunting	H/A	<−2 SD		Short stature	H/A	<5th percentile
Adult ≥ 20 y Underweight: BMI < 18.5 kg/m^2^							
Overnutrition							
0–5 y	Overweight	W/L or W/H or BMI	>+2 SD and ≤+3 SD	0–2 y	Overweight and obesity	Recommend using WHO growth standards	
	Obesity	W/L or W/H or BMI	>+3 SD				
5–19 y	Overweight	BMI	>+1 SD and ≤+2 SD	2–19 y	Overweight	BMI	85th to 94th percentile
	Obesity	BMI	>+2 SD		Obesity	BMI	≥95th percentile
Adults ≥ 20 y Overweight: BMI 25.0–29.9 kg/m^2^ Obesity: BMI ≥ 30 kg/m^2^							

Abbreviations: BMI, body mass index; CDC, Centers for Disease Control and Prevention; H/A, height for age; L/A, length for age; SD, standard deviation; W/A, weight for age; W/L, weight for length; W/H, weight for height; WHO, World Health Organization; y, years.

**Table 3:** Advantages and disadvantages of anthropometry and body composition measurement [20,46,47].

**Anthropometric Methods**	**Advantages**	**Disadvantages**
Weight, height, BMI	Inexpensive Simple Suitable for large-scale studies	Unable to distinguish fat and fat-free mass tissue Unreliable for patients with large tumors
Waist measurement	Inexpensive Simple Surrogate estimate of trunk and visceral fat	Operator dependent Unreliable for patients with abdominal masses
MUAC	Inexpensive Simple More accurate in specific conditions (e.g., large tumor mass, edema)	Operator dependent No standard cut-offs for overweight or obesity
Skinfold thickness	Inexpensive Simple Can assess total body fat store	Operator dependent Requires instruments and specific equation assumptions
**Advanced Methods**	**Advantages**	**Disadvantages**
BIA	Inexpensive Quick and non-invasive Simple and reproducible Reference values are available	Indirect method Limited by hydration status Specific equation needed for each population Limited availability in LMICs
DXA	Able to differentiate fat, lean mass, and bone tissue Quick and non-invasive Low radiation exposure Well tolerated for repeated measurement High precision and accuracy Reference values available	Relatively high cost Variability of instrument calibration, procedure Inability to discriminate type of fat (visceral, subcutaneous, or intramuscular) Limited availability in LMICs
CT	Useful for evaluating specific body compartments, such as visceral/subcutaneous/adipose tissue Can use imaging for disease evaluation in patients with abdominal lesions	Radiation exposure High cost Limited population control
MRI	Useful for evaluating specific body compartments, such as visceral/subcutaneous/adipose tissue Can use imaging for disease evaluation in patients with abdominal lesions Provides soft tissue information: muscle edema, inflammation, atrophy, and fatty infiltration	Long study time Requires sedation of younger children High cost Limited population control

Abbreviations: BIA, bioelectrical impedance; BMI, body mass index; CT, computed tomography; DXA, dual-energy x-ray absorptiometry; LMICs, low-/medium-income countries; MUAC, mid-upper arm circumference; MRI, magnetic resonance imaging.

**Table 4:** Summary of recommendations on nutritional assessment of children and adolescents with cancer in settings with differing access to resources [35,36,37,59].

	Limited Access	Partial Access	Full Access
History taking	Use screening tools to identify patients at risk, e.g., use SCAN for every patient	Complete history taking for every patient	Complete history taking for every patient Comprehensive dietary assessment methods such as FFQ
Physical examination	Identify specific signs and symptoms of micronutrient deficiencies, especially in patients with suspected severe malnutrition	Complete physical examination for every patient	Complete physical examination for every patient
Anthropometric measurements	Wt, Ht, BMI MUAC cut-off for age	Wt, Ht, BMI, MUAC, WC, TSFT Use percentiles or Z-scores based on age and sex Include waist measurement for patients at risk of overweight/obesity Longitudinal growth curve plot	Wt, Ht, BMI, MUAC, WC, TSFT Use percentiles or Z-scores based on age and sex Include waist measurement for patients at risk of overweight/obesity Longitudinal growth curve plot
Biochemistry evaluation	Complete blood count Basic metabolic panel and albumin Prioritize investigations in patients with severe malnutrition (aim to prevent refeeding syndrome)	Complete blood count Comprehensive metabolic panels Specific micronutrient work-up based on history assessment and clinical signs	Complete blood count Comprehensive metabolic and micronutrient panels Screen all patients to identify those at risk
Advanced body composition methods	Not indicated	BIA Prioritized for patients at risk for conditions such as abnormal BMI, sarcopenia, osteopenia, or abnormal cardiometabolic profile or for those receiving treatments that increase the risk of cardiometabolic syndrome (e.g., corticosteroids, cranial radiation)	Advanced body composition methods such as DXA Consider using CT or MRI for disease evaluation or staging of abdominal tumors Screening at baseline to improve accuracy to diagnose specific conditions, (e.g., obesity, visceral organ adiposity, osteopenia, or sarcopenia) Longitudinal monitoring for patients at risk
Follow-up	Follow-up assessments of at-risk patients	History taking, physical examination, basic biochemistry evaluation as routine	History taking, physical examination, basic biochemistry evaluation as routine Longitudinal laboratory and advanced body composition monitoring for at-risk patients

Abbreviations: BIA, bioelectrical impedance; BMI, body mass index; CT, computed tomography; DXA, dual-energy x-ray absorptiometry; FFQ, food-frequency questionnaire; Ht, height; MRI, magnetic resonance imaging; MUAC, mid-upper arm circumference; SCAN, the nutrition screening tool for childhood cancer; TSFT, triceps skinfold thickness; WC, waist circumference; Wt, weight.

## Data Availability

No new data were created or analyzed in this study. Data sharing is not applicable to this article.

## Supplementary Materials

The following supporting information can be downloaded at: https://www.mdpi.com/article/10.3390/cancers18050873/s1, Table S1. Prevalence of undernutrition and overnutrition in children with cancer in low-, middle-, and high-income countries; Table S2. Clinical signs of nutritional deficiencies; Table S3. Clinical studies of anthropometric body composition analysis in pediatric patients with cancer at diagnosis; Table S4. Clinical studies of body composition as measured with anthropometric methods and advanced techniques in pediatric patients with cancer and in pediatric cancer survivors.
