# Supplementary material for: Nutritional Assessment of Children and Adolescents with Cancer in Various Resource Settings
Source: Cancers (Basel). 2026 Mar 8;18(5):873. doi: 10.3390/cancers18050873 (PMC12984784; doi:10.3390/cancers18050873)
Supplement: Supplementary file 1 [file cancers-18-00873-s001.zip › cancers-4137507-supplementary.pdf]

Supplementary Table S1. Prevalence of undernutrition and overnutrition in children with cancer in low-, middle-, and high-income countries.

	Study, country	*N*	Age (y)	Tumor type (%)	Anthropometric classification reference	Methods	Prevalence of nutritional status
**Low-income countries**	**Huibers et al.**[4]<br>(Malawi)	463	<19	Hematologic malignancy (52.1)<br>Solid tumor (47.9)	WHO	W/H/BMI	At diagnosis<br>Underweight: 30.5%
	**Lifson et al.**[5]<br>(South Africa)	76	0.5–13	Wilms tumor (100)	WHO	BMI	At diagnosis<br>Underweight: 29.3%
**Middle-income countries**	**Muhammad et al.**[6]<br>(Pakistan)	384	1–16	Hematologic malignancy (69.8)<br>Solid tumor (30.2)	WHO	BMI	At diagnosis<br>Underweight: 45.6%
	**Yaprak et al.**[12]<br>(Turkey)	81	<18	Solid tumor (43.2)<br>Lymphoma (30.9)<br>Other (25.9)	Turkey reference	BMI	At diagnosis<br>Underweight: 23.5%
	**Sasse et al.**[7]<br>(Brazil)	99	<19	Solid tumor (100)	WHO	BMI	At diagnosis<br>Underweight: 8.1%<br>Overweight or obesity: 14.1%
	**Sala et al.**[13]<br>(Central America; Costa Rica, Dominican Republic, El Salvador, Guatemela, Honduras, Nicaragua, Panama)	1787	1–18	Hematologic malignancy (71.4)<br>Solid tumor (22.3)<br>Other (6.3)	CDC	BMI	At diagnosis<br>Underweight: 28.0%
	**Shah et al.**[14]<br>(India)	1187	2–15	Hematologic malignancy (67.9)<br>Solid tumor (25.9)<br>Other (6.2)	WHO	BMI	At diagnosis<br>Underweight: 39.9%
**High-income countries**	**Collins et al.**[8]<br>(Canada)	99	≤18	Hematologic malignancy (87.9)<br>Solid tumor (12.1)	CDC	BMI	At diagnosis<br>Underweight: 9.0%<br>Overweight: 7.0%
	**Orgel et al.**[9]<br>(USA, Canada, Australia, and New Zealand)	2008	1–20	ALL (100)	CDC	W/H (<2 years)<br>BMI (2–20 years)	At diagnosis<br>Underweight: 5.8%

						Obesity: 13.9%
**Triarico et al.**[10] (Italy)	126	3–17	Solid tumor (73.0)<br>Hematologic malignancy (27.0)	CDC	BMI	At diagnosis<br>Underweight: 17.4%
**Browne et al.**[15] (USA)	372	2–18	ALL (100)	CDC	BMI	At diagnosis<br>Underweight: approximately 7.0%<br>Overweight or obesity: 25.5%<br>At off-therapy<br>Underweight: approximately 5.0%<br>Overweight or obesity: 41.6%
**Iijima et al.**[11] (USA)	227	0–21	AML (100)	CDC	W/L (<2 years)<br>BMI (2–20 years)	At diagnosis<br>Underweight: 9.7%<br>Overweight or obesity: 20.7%<br>At 1-year off-therapy<br>Underweight: 2.0%<br>Overweight or obesity: 42.6%

Abbreviations: ALL, acute lymphoblastic leukemia; AML, acute myeloid leukemia; BMI, body mass index; CDC, Centers for Disease Control and Prevention; H/A, height for age; NCHS, National Center for Health Statistics percentiles; W/A, weight for age; W/H, weight for height; WHO, World Health Organization

Supplementary Table S2. Clinical signs of nutritional deficiencies.

Examination site	Clinical sign	Nutritional deficiency
Hair	Hair change, thin, sparse, depigmentation	Protein/energy, zinc, copper, iron, niacin, biotin, selenium
Subcutaneous tissue	Edema<br>Loss of subcutaneous fat	Protein, sodium<br>Energy
Skin	Dry, flaky rough, desquamating dermatitis<br>Petechiae, bruising<br>Periorificial dermatitis	Essential fatty acids, vitamin B<br>Vitamin A, vitamin C<br>Zinc
Eyes	Pale conjunctivae<br>Dry conjunctiva, keratomalacia	Iron, vitamin B, folate<br>Vitamin A
Lips	Angular stomatitis, cheilosis	Vitamin B
Tongue	Glossitis, color change	Iron, vitamin B
Gums	Gingivitis, bleeding easily	Vitamin C
Neck	Thyroid enlargement	Iodine
Nails	Spoon shape, koilonychia	Iron, zinc, copper
Muscles	Wasting	Protein/energy, zinc
Neurological	Peripheral neuropathy	Vitamin B12

Supplementary Table S3. Clinical studies of anthropometric body composition analysis in pediatric patients with cancer at diagnosis.

Study	Design	*N*	Age (y)	Diseases (%)	Parameters	Reference/criteria	Results
**Huibers et al.**[4]<br>(Malawi)	Prospective cohort study	463	<19	Hematologic malignancy (52.1)<br>Solid tumors (47.9)	Wt, Ht, BMI, MUAC	**WHO reference**[38,53]<br>**Age < 5 y**<br>**Moderate undernutrition:**<br>WFA or WFH or WFA Z-score < –3 to –2 and/or MUAC > 11.5 to < 12.5 cm<br>**Severe undernutrition:**<br>WFA or WFH or WFA Z-score ≤ –3 and/or MUAC ≤ 11.5 cm<br><br>**Age 5–9 years**<br>**Moderate undernutrition:**<br>WFA or BMI Z-score –2 to < –3 and/or MUAC > 13 to < 15.5 cm<br>**Severe undernutrition:**<br>WFA or BMI Z-score ≤ –3 and/or MUAC ≤ 13 cm<br><br>**Age ≥ 10 years**<br>**Moderate undernutrition:**<br>BMI Z-score –2 to < –3 and/or MUAC > 16 to ≤ 18.5 cm<br>**Severe undernutrition:**<br>BMI Z-score ≤ –3 and/or MUAC ≤ 16 cm	- At diagnosis, prevalence of moderate undernutrition was 13.7% (according to W/H or BMI), 15.3% (according to W/A), 27.9% (according to MUAC)<br><br>- At diagnosis, prevalence of severe undernutrition was 16.8% (according to W/H or BMI), 12.2% (according to W/A), 42.7% (according to MUAC)<br><br>- Severe malnutrition and age < 5 years were associated with increased risk of mortality, with HR of 1.6 (95% CI, 1.1–2.3, *P* = 0.012) and 1.6 (95% CI, 1.1–2.3, *P* = 0.016), respectively.
**Lifson et al.**[5]<br>(South Africa)	Prospective cohort study	76	0.5–13	Wilms tumor (100)	Wt, Ht, BMI, TSFT, MUAC, albumin	**WHO reference**[38] (for BMI)<br>**Frisancho reference**[56] (for TSFT, MUAC)<br><br>**WHO criteria for malnutrition**[38,53]<br>**Mild underweight:**<br>WFA/HFA/BMI/MUAC/TSFT Z-score < –1	- At diagnosis, prevalence of undernutrition was<br>35.2% (according to WFA)<br>38.7% (according to HFA)<br>29.3% (according to BMI)<br>79.7% (according to MUAC)<br>71.8% (according to TSFT)

						**Moderate underweight:**<br>WFA/HFA/BMI/MUAC/TSFT Z-score < –2<br>**Severe underweight:**<br>WFA/HFA/BMI/MUAC/TSFT Z-score < –3<br><br>**Modified AHOPCA criteria for malnutrition**[57]<br>**Moderate undernutrition:**<br>- MUAC and TSFT 5th to 10th percentile OR<br>- MUAC or TSFT < 5th percentile OR<br>- Albumin < 3.5 g/dL<br>**Severe undernutrition:**<br>- MUAC and/or TSFT < 5th percentile OR<br>- Albumin < 3.2 g/dL	30.3% (according to albumin)<br>56.9% (according to MUAC and TSFT)<br>66.7% (according to MUAC, TSFT, and albumin)<br>- No significant association between nutritional status and disease stage<br>- No significant association between malnutrition on admission and mortality at 2 years after diagnosis
**Sasse et al.**[7]<br>(Brazil)	Prospective cohort study	99	<19	Solid tumor (100)	Wt, Ht, BMI, TSFT, MUAC	**WHO reference**[38] (for BMI)<br>**- Undernutrition:** HFA or BMI Z-score < –2<br>**- Overweight:** BMI Z-score > +2<br>**- Obesity:** BMI Z-score > +3<br><br>**Frisancho reference**[56] (for MUAC)<br>**- Undernutrition:** MUAC ≤ 5th percentile<br>**- Overweight/obesity:** MUAC ≥ 85th percentile	- At diagnosis, prevalence of undernutrition was<br>8.1% (according to BMI)<br>29.3% (according to BMI and MUAC)<br><br>- At diagnosis, prevalence of overweight/obesity was<br>14.1% (according to BMI)<br>21.2% (according to BMI and MUAC)<br><br>- At 3 and 6 months after diagnosis, prevalence of undernutrition decreased to 27.3% and 18.8%, respectively (according to BMI and MUAC).<br>- At 3 and 6 months after diagnosis, prevalence of overweight/obesity increased to 23.9% and 27.5%, respectively (according to BMI and MUAC).
**Sala et al.**[13]<br>(Central America)	Prospective cohort study	1787	1–18	Hematologic malignancy (71.4)<br>Solid tumor (22.3)<br>Other (6.3)	Wt, Ht, BMI, IBW, MUAC, TSFT, albumin	**CDC reference**[39] (Wt, Ht, BMI)<br>**Frisancho reference**[56]<br>(for TSFT, MUAC)<br><br>**AHOPCA criteria for malnutrition**[57]	- At diagnosis, prevalence of undernutrition was<br>28.0% (according to BMI)<br>63.5% (according to MUAC and TSFT)<br>66.9% (according to MUAC, TSFT, and IBW)<br>77.2% (according to MUAC, TSFT, and albumin)

						**Moderate acute malnutrition:**<br>- MUAC 5th to 10th percentile OR<br>- TSFT 5th to 10th percentile OR<br>- Albumin 3.2 to 3.5 g/dL<br>**Severe acute malnutrition:**<br>- MUAC < 5th percentile OR<br>- TSFT < 5th percentile OR<br>- Albumin < 3.2 g/dL	- The abandonment rate was significantly higher in patients with ALL or solid tumors who had malnutrition than in those without malnutrition ($P < 0.043$).<br>- Increased mortality was observed in patients with solid tumors with severe malnutrition ($P < 0.001$).<br>- Patients with solid tumors or lymphoma who had severe malnutrition had significantly worse 2-year EFS when compared with those with adequate or moderately depleted nutrition ($P \leq 0.020$).
**Shah et al.**[14]<br>(India)	Retrospective observational study	1187	2–15	Hematologic malignancy (67.9)<br>Solid tumor (25.9)<br>Other (6.2)	BMI, TSFT, MUAC, AMC, albumin	**WHO reference**[38] (for BMI)<br>**- Undernutrition:** BMI Z-score < –2<br>**- Overweight:** BMI Z-score > +2<br>**- Obesity:** BMI Z-score > +3<br><br>**Frisancho reference**[56] (for TSFT, MUAC, AMC)<br>**- Undernutrition:**<br>MUAC/TSFT/AMC ≤ 10th percentile<br>**- Overweight/obesity:**<br>MUAC/TSFT/AMC ≥ 85th percentile<br><br>**St. Jude Children's Research Hospital algorithm**[13,57]<br>**Moderate undernutrition:**<br>- MUAC 5th to 10th percentile OR<br>- TSFT 5th to 10th percentile OR<br>- Albumin 3.2 to 3.5 g/dl<br>**Severe undernutrition:**<br>- MUAC < 5th percentile OR<br>- TSFT < 5th percentile OR<br>- Albumin < 3.2 g/dl	- At diagnosis, prevalence of undernutrition was<br>39.9% (according to BMI)<br>56.9% (according to TSFT)<br>68.7% (according to AMC)<br>75.7% (according to MUAC)<br>82.5% (according to TSFT and MUAC)<br>84.0% (according to TSFT, MUAC, and albumin)<br>84.5% (according to TSFT, MUAC, and BMI)
**Villanueva et al.**[18]<br>(Guatemala)	Retrospective cohort study	1060	0–18	Hematologic malignancy (62.5)	MUAC, TSFT, albumin	**WHO reference**[53] (children ≤ 5 y)<br>**Frisancho reference**[56] (children > 5 y)	- At diagnosis, prevalence of undernutrition was<br>24.0% (according to TSFT)

				Solid tumor (37.5)		**Moderate undernutrition:**<br>- MUAC 5th to 10th percentile OR<br>- TSFT 5th to 10th percentile OR<br>- Albumin 3.2 to 3.5 g/dL<br>**Severe undernutrition:**<br>- MUAC < 5th percentile OR<br>- TSFT < 5th percentile OR<br>- Albumin < 3.2 g/dL	44.0% (according to MUAC)<br>72.0% (according to TSFT, MUAC, and albumin)<br>- Patients with leukemia and lymphoma had a significantly greater risk of undernutrition at diagnosis when compared with patients with brain tumors, with odds ratios of 6.08 (95% CI, 1.74–28.28; *P* = 0.008) for leukemia and 4.83 (95% CI, 1.33–23.03; *P* = 0.03) for lymphoma.<br>- Lower socioeconomic status at diagnosis was significantly associated with a decline in nutritional status (*P* < 0.001).
**Revuelta Iniesta et al.**[51]<br>(Scotland)	Prospective cohort study	82	<18	Hematologic malignancy (58.5)<br>Solid tumor (41.5)	Wt, Ht, BMI, TSFT, MUAC, AMA, AFA	**UK reference**[51] (for BMI)<br>**- Undernutrition:**<br>BMI Z-score ≤ −2 or 2.3rd percentile<br>**- Overweight:**<br>BMI Z-score ≥ +1.05 or 85th percentile<br>**- Obesity:**<br>BMI Z-score ≥ +1.63 or 95th percentile<br><br>**Frisancho reference**[56]<br>(for TSFT, MUAC, AMA, AFA)<br>**- Undernutrition:**<br>MUAC/TSFT/AMA/AFA ≤ 5th percentile<br>**- Overweight:**<br>MUAC/TSFT/AMA/AFA ≥ 85th percentile<br>**- Obesity:**<br>MUAC/TSFT/AMA/AFA ≥ 95th percentile	- Prevalence of undernutrition was highest at diagnosis of ranged between 13.6% of BMI, 12.7% of MUAC and 14.7% of TSFT.<br>- According to BMI, no undernourished patients were detected at 9, 12, and 18 months; however, MUAC and TSFT identified 3%–6% as undernourished.<br>- Overweight patients were identified at 7%–21% during the study period, peaking at 30 and 36 months, whereas the prevalence of obesity was lowest at diagnosis and highest at 30 months.<br>- Prevalence of overweight by BMI and TSFT was similar at baseline, 3, 6, 18, and 24 months, but TSFT detected more cases at 9 and 12 months, with differences of 7% and 21%, respectively.<br>- The multilevel growth model revealed a statistically significant increase in the mean BMI percentile from 0 to 3 months and from 0 to 18 months. In contrast, there was a significant decrease in fat-free mass, as measured by arm anthropometry, from 0 to 3 months.
**Yaprak et al.**[12]<br>(Turkey)	Prospective cohort study	81	<18	Solid tumor (43.2)<br>Lymphoma (30.9)<br>Other (25.9)	Wt, Ht, BMI, TSFT, MUAC, AA, AMA, AFA	**Turkey** (all variables)<br>**ASPEN classification**[12]<br>**- Mild/at risk of malnutrition:**<br>WFH/BMI/MUAC/WFA/arm anthropometry Z-scores −1 to −1.99	- At diagnosis, prevalence of undernutrition according to BMI, MUAC, TSFT, AMA, AFA, AA, MUAC/TSFT, and MUAC/TSFT/BMI was 23.5%, 27.2%, 21%, 16%, 19.8, 21%, 35.8%, and 40.7%, respectively.<br>- At diagnosis, malnutrition rates defined by BMI did

						**- Moderate malnutrition:**<br>Z-scores −2 to −2.99<br>**- Severe malnutrition:**<br>Z-scores ≤ −3	not differ according to clinical and pathological characteristics.<br>- The prevalence of malnutrition defined by MUAC/TSFT was higher in patients younger than 5 years (*P* = 0.03), those with abdominal tumors (*P* = 0.03), and those with advanced disease (*P* < 0.001).<br>- Patients with undernutrition had worse 5-year overall survival.

Abbreviations: AA, arm area; AFA, arm fat area; Alb, albumin; AMA, arm muscle area; AMC, arm muscle circumference; BMI, body mass index; HFA, height for age; Ht, height; IBW, ideal body weight; MUAC, mid-upper arm circumference; NCHS, National Center for Health Statistics; Pt, patient; TSFT, triceps skinfold thickness; WFA, weight for age; WFH, weight for height; WHO, World Health Organization; Wt, weight.

Supplementary Table S4. Clinical studies of body composition as measured with anthropometric methods and advanced techniques in pediatric patients with cancer and in pediatric cancer survivors

Study	Design	*N*	Age (y)	Diseases (%)	Anthropometric methods (reference)	Advanced body composition measurements	Time of measurement	Results
**Chincesan et al.**[52] (Romania)	Prospective cohort study	146	5-18	43 cancer cases and 103 healthy controls<br>Leukemia (51.2)<br>Lymphoma (23.2)<br>Solid tumor (25.6)	Wt, Ht, BMI, TSFT, MUAC	BIA	Diagnosis	- Significant lower values Z-scores for Wt, Ht, BMI, MUAC, and TSFT were observed in children with cancer when compared to healthy children.<br>- BIA measurements also showed that children with cancer had significantly lower fat mass when compared to controls.<br>- There was a significantly positive correlation between BMI and FM; BMI and TSFT; MUAC and muscle mass; and MUAC and FFM.
**Siviero-Miachon et al.**[78] (Brazil)	Cross-sectional study	56	15-24	ALL (100)<br>Receiving CRT (44.6)<br>Not receiving CRT (55.4)	BMI, WC, HC, WHR, WHtR	DXA<br>Abdominal CT	Cancer survivors	- There were no differences in BMI, BMI Z-score, WC, WHR, or WHtR between CRT recipients and non-CRT recipients. However, when considering the CT and DXA variables, the CRT group had significantly higher adipose tissue and fat mass indices when compared with the non-CRT group.
**Barr et al.**[50] (Canada)	Prospective cohort study	99	NA	Hematologic malignancy (74.0)<br>Extracranial solid tumor (10)<br>Other (16)	TSFT, MUAC	DXA<br>(lean body mass)	Diagnosis	- There was a positive correlation between MUAC and lean body mass.<br>- There was a weak correlation between TSFT and fat mass.
**Revuelta Iniesta et al.**[51] (Scotland)	Prospective cohort study	82	<18	Hematologic malignancy (58.5)<br>Solid tumor (41.5)	BMI, TSFT, MUAC, AMA, AFA	BIA	Diagnosis and during treatment	- There was a statistically significant increase in mean BMI percentile from 0–3 months and from 0–18 months. Simultaneously, there was a significant decrease in the FFM as measured by using BIA at 0–3 months and at 0–9 months.<br>- During the study, the decrease in the FFM percentage as measured using MUAC and AMA showed a trend similar to that calculated by BIA. Similarly, the increase in FM percentage, assessed using TSFT and AFA, consistently aligned with the trend in BIA measurements at all time points.

**Karlage et al.**[30] (USA)	Retrospective cohort study	1361	>18	Hematologic malignancy (71.6)<br>Solid tumor (26.1)<br>Other (2.3)	BMI, skinfold thickness, WHtR	DXA	Cancer survivors	- There were significant positive correlations between percent body fat, as measured by skinfolds and by DXA, in both male and female participants. These were better than the correlations between BMI and percent body fat as measured by DXA and between WHtR and percent fat as measured by DXA.<br>- WHtR was the most sensitive method for detecting obesity among male survivors, with a sensitivity of 90.2%. Among female survivors, WHtR and skinfolds were more sensitive than BMI for detecting obesity, with sensitivities of 75.6%, 73.7%, and 51.3%, respectively.<br>- Overall, specificity was high for all measures except WHtR among male survivors.
**Brinksma et al.**[76] (Netherlands)	Prospective cohort study	133	0.1-17.7	Hematologic malignancy (39.8)<br>Solid tumor (33.1)<br>Brain tumor (27.1)	BMI	BIA	Diagnosis and 3, 6, and 12 months	- At diagnosis, 17.2% of patients were identified as undernourished based on FFM < −2 SDS, whereas BMI detected undernutrition in only 8.3% of patients. Additionally, overnourishment was diagnosed in 10.9% based on FM > 2 SDS, as compared with 4.5% detected by BMI.<br>- BMI and FM significantly increased within 3 months and continued to rise in the following months, whereas FFM, which was low at diagnosis, remained low.
**Blijdorp et al.**[77] (Netherlands)	Retrospective cohort study	422	21.9-32.7	Hematologic malignancy (71.7)<br>Solid tumor (20.3)<br>Other (8.0)	BMI, WHR	DXA (total fat percentage based on Dutch reference)	Cancer survivors	- Half of the patients classified as obese by DXA based on total fat percentage were misclassified as non-obese according to BMI criteria, whereas 31% were misclassified based on WHR criteria.<br>- Male survivors had a significantly lower BMI at their first visit, whereas female survivors had a significantly higher BMI compared to reference values. However, the fat percentage was significantly higher and the lean body mass was significantly lower in survivors of both sexes when compared with Dutch references.<br>- Cut-off points for WHR, as defined by the WHO, were more sensitive than BMI for detecting a high total fat percentage in survivors.

**Ritz et al.**[70]<br>(Spain)	Prospective cohort study	101	1-15	Neuroblastoma	Wt, Ht, BMI<br>(WHO)	CT or MRI	Before surgery<br>(Median time after diagnosis, 7 months; IQR, 4–12 months)	- tPMA Z-scores at L4–5 showed a moderate correlation with WFA Z-scores (r = 0.54; 95% CI, 0.38–0.66; $P < 0.001$) and a poor correlation with HFA (r = 0.35; 95% CI, 0.17–0.52; P = 0.001) and BMI Z-scores (r = 0.38; 95% CI, 0.20–0.54; P = 0.001).<br>- Sarcopenia before surgery was not associated with hospital stay or time in ICU after surgery.<br>- Sarcopenia before surgery had a sensitivity of 0.82 (95% CI, 0.62–0.93) and a specificity of 0.48 (95% CI, 0.36–0.61) in predicting 5-year survival, with hazard ratios of 4.18 (95% CI, 1.01–17.26).<br>- Sarcopenia, age at diagnosis, unfavorable tumor histology, and NB2004-HR chemotherapy were identified as predictors of poor outcomes.
**Romano et al.**[71]<br>(Italy)	Prospective cohort study	22	Median age 10.5 (IQR, 6.6-15.1)	Sarcoma<br>(EWS 66%, RMS 28.6%)	BMI<br>(No reference data available)	CT	At diagnosis and after 12 months of treatment	- Over half of the patients had sarcopenia at diagnosis, as detected by CT, but all of them had a normal BMI.<br>- BMI Z-score and tPMA Z-score significantly decreased after 12 months of treatment.<br>- Risk factors associated with poor overall survival were the presence of metastasis, the absence of surgery, changes to the prognostic nutritional index (PNI), and a reduction in tPMA of more than 25%.
**Joffe et al.**[72]<br>(USA)	Retrospective cohort study	39	Median age 11 (range, 1.33-20.0)	Solid tumor<br>RMS (41)<br>EWS (20.5)<br>WT (20.5)<br>OS (18)	BMI percentile<br>(CDC, WHO)	CT	At diagnosis and after treatment for 6–14 weeks (mean timing between image, 12.1 weeks; SD, 3.3)	- There was a poor association between BMI and body composition parameter as determined using CT.<br>- There was a strong correlation between body composition variables at T12–L1 and L3.<br>- There was a significant decrease in skeletal muscle and lean tissue and a significant increase in visceral adipose tissue after treatment, as measured using CT.<br>- A risk factor associated with muscle mass loss was age >12 years.

**Tram et al.**[79] (USA)	Retrospective cohort study	110	Mean age 13.5 ± 0.4 (range, 2.2-20.8)	NHL (100)	BMI (CDC)	CT	At diagnosis and At first follow-up (4 to 20 weeks after treatment initiation)	- BMI percentage did not change from diagnosis to first follow-up, but increases in VAT and SAT, along with skeletal muscle decline, were detected by CT.<br>- Male sex, age <12 years, NHL, and advanced stage of disease were risk factors for gaining more adipose tissue and losing more muscle during the first cycle of treatment.
**Suwannaying et al.**[25] (USA)	Prospective cohort study	189	2-18	ALL (100)	BMI (CDC)	Quantitative CT	At diagnosis and off-therapy	- BMI Z-score was positively correlated with VAT, SAT, and TAT but negatively correlated with LT/TAT and VAT/SAT.<br>- At off-therapy, BMI Z-score, SAT, VAT, and TAT were higher, whereas LT, LT/TAT, and VAT/SAT were lower when compared to their values at diagnosis.<br>- Older age (≥10 years) at diagnosis was associated with increased adipose tissue. Female sex was associated with lower lean tissue, and Black patients had lower VAT when compared with White patients.

Abbreviations: AFA, arm fat area; AMC, arm muscle circumference; ASCT, autologous stem cell transplantation; BIA, bioelectric impedance analysis; BMI, body mass index; CRT, cranial radiation; CT, computed tomography; DXA, dual-energy x-ray absorptiometry; EWS: Ewing sarcoma; FFM, fat-free mass; FM, fat-mass; Ht, height; IQR, interquartile range; MUAC, mid-upper arm circumference; IMAT, intramuscular adipose tissue; NHL: non-Hodgkin lymphoma; RMS: rhabdomyosarcoma; SAT, subcutaneous adipose tissue; TAT, total adipose tissue; tPMA: psoas muscle area; TSFT, triceps skinfold thickness; VAT, visceral adipose tissue; Wt, weight; WHR, waist-to-hip ratio; WHtR, waist-to-height ratio.
